# Gallic acid exerts therapeutic effects on sciatica by reducing inflammatory responses through the regulation of NOX4-mediated oxidative stress

**DOI:** 10.3389/fimmu.2026.1748652

**Published:** 2026-05-13

**Authors:** Zenni Chen, Zifeng Zhuang, Shaozi Lin, Yuting Hao, Xiaokang Xie, Hongling Chen, Lan Lin, Yixuan Li, Lili Fan, Shiquan Chang, Guoping Zhao, Di Zhang

**Affiliations:** 1 College of Traditional Chinese Medicine, Jinan University, Guangzhou, China; 2 Guangzhou Medical University Affiliated Traditional Chinese Medicine Hospital, Guangzhou, China; 3 Guangzhou Key Laboratory of Formula-Pattern of Traditional Chinese Medicine, School of Traditional Chinese Medicine, Jinan University, Guangzhou, Guangdong, China

**Keywords:** gallic acid, macrophage polarization, neuroinflammation, oxidative stress, sciatica

## Abstract

**Background:**

Sciatica causes severe pain and impaired mobility. Neuroinflammation is involved in the development of sciatica.

**Purpose:**

This study aimed to explore whether Gallic acid (GA) reduces neuroinflammation to relieve sciatica by regulating NOX4-mediated oxidative stress.

**Methods:**

After scRNA-seq analysis was performed, 32 SD (Sprague-Dawley) rats were randomly divided into 4 groups: sham operation, chronic constriction injury (CCI), CCI+mecobalamin, and CCI+GA groups. We conducted behavioral tests, ELISA, western blotting, and immunofluorescence analysis. In cell experiments, we conducted ROS measurement, flow cytometry, PCR, and western blotting.

**Results:**

scRNA-seq analysis revealed that gene signatures related to the “inflammatory response” and “oxidative stress” were significantly enriched, with higher module scores observed specifically in M1 macrophages. In RAW264.7 cells, LPS stimulation significantly increased ROS generation and MDA levels and upregulated the expression of M1 macrophage markers, including IL-1β, iNOS, TNF-α, and CD32. In addition, LPS increased the protein expression of NOX4 and inflammatory mediators (TNF-α, IBA-1, IL-1β, COX-2, and iNOS) while reducing the levels of ATF4 and p-Nrf2. GA treatment reduced ROS generation and MDA levels; downregulated the mRNA expression of IL-1β, iNOS, TNF-α, and CD32; and increased CD206 mRNA expression. Similarly, GA decreased the protein levels of NOX4, TNF-α, IBA-1, IL-1β, COX-2, and iNOS but restored the expression of ATF4 and p-Nrf2. In CCI rats, GA significantly attenuated thermal hyperalgesia from Day 7 to Day 21, with thermal withdrawal thresholds recovering toward sham control levels. CCI markedly increased IL-8, COX-2, TNF-α, TGF-β, IL-6, and IL-1β levels in the sciatic nerve; increased IBA-1/CD32 coexpression; decreased IBA-1/CD206 coexpression; and markedly disrupted sciatic nerve architecture. These pathological changes were accompanied by elevated expression of IBA-1, NOX4, IL-1β, and iNOS, together with reduced ATF4 and p-Nrf2 levels. Notably, GA treatment largely reversed these CCI-induced alterations.

**Conclusion:**

GA alleviated sciatica in a rat model, possibly through its ability to promote the polarization of proinflammatory M1 macrophages toward anti-inflammatory M2 macrophages via the regulation of NOX4-mediated oxidative stress.

## 1 Introduction

Sciatica refers to radiating pain in the lower limb resulting from inflammation or compression of lumbosacral nerve roots (1), causing severe pain. Although most patients recover spontaneously or respond to conservative treatments, a subset of patients experience persistent or recurrent symptoms that may require further intervention (2). Among patients with chronic sciatica, 45% fail to achieve meaningful improvement after one year (3) and 34% continue to experience chronic pain beyond two years (4). The National Institute for Health and Care Excellence (NICE) indicated that gabapentinoids, other antiepileptics, oral corticosteroids or benzodiazepines should not be used to manage sciatica, as there is no overall evidence of benefit but there is evidence of harm (4). Despite the widespread use of paracetamol, nonsteroidal anti-inflammatory drugs, and opioids for sciatica, clinical practice guidelines provide inconsistent guidance about these drugs, reflecting limited evidence of benefit and concerns about harm (5). Ongoing debate about the efficacy and safety of available treatments complicates the management of chronic sciatica, creating an urgent imperative to develop new pharmacological options.

Chronic pain has been widely reported to be relieved with the inhibition of macrophage activation (6–9). Macrophages can differentiate into classically activated (M1) and alternatively activated (M2) phenotypes (10, 11). The M1 phenotype primarily mediates proinflammatory activities by producing a range of inflammatory cytokines that perpetuate and worsen pain (12), whereas the M2 phenotype is centrally involved in anti-inflammatory actions, facilitating tissue repair and the resolution of inflammation (13). The immediate mechanical insult to the sciatic nerve provokes an increase in reactive oxygen species (ROS) production (14, 15), driving inflammatory and immune responses (16). The inhibition of ROS production can relieve mechanical allodynia (17). NOX4 was an important enzymatic source of reactive oxygen species, whereas nuclear factor erythroid 2–related factor 2 (Nrf2) was a central transcription factor that coordinated antioxidant and anti-inflammatory responses (18). Besides, ATF4 was involved in inflammatory macrophage activation (19). ATF4 had been implicated in cellular stress adaptation and may functionally interact with Nrf2 under oxidative conditions (20). Therefore, modulation of macrophage polarization and the NOX4-related oxidative stress pathway may represent an important mechanism for alleviating sciatica.

Gallic acid (GA) has been shown to possess anti-inflammatory, antioxidant, and analgesic properties (21). GA exerts its antioxidant effects by reducing ROS production (22, 23). GA enhances the expression of Nrf2 and attenuates ROS generation to mitigate the inflammatory response induced by lipopolysaccharide (LPS) (24). In this study, we investigated whether NOX4-related oxidative stress and macrophage polarization contribute to the pathogenesis of sciatica and whether GA can modulate these processes to improve pain-related behaviors. Using a chronic constriction injury (CCI) rat model and LPS-stimulated RAW264.7 macrophages, we aimed to (1) determine whether GA has analgesic effects on CCI rats; (2) elucidate the effects of GA on the NOX4/Nrf2 signaling axis, oxidative stress markers, and M1/M2 macrophage polarization; and (3) assess the requirement of NOX4 for the GA-mediated regulation of macrophage function. Collectively, these findings may help identify mechanistic targets for the development of novel therapies for sciatica.

## 2 Materials and methods

### 2.1 Bioinformatics analysis of single cell sequencing

Single-cell RNA sequencing data from the rat sciatic nerve model (GSE216665) (25) were processed using R (v4.4) and Seurat (v5.0.1). Highly variable genes were identified with the FindVariableFeatures function in Seurat, employing the “vst” method, and the top 2,000 genes were selected for further analysis (10, 26). Principal component analysis (PCA) was carried out with the RunPCA function using default settings, and the first 30 principal components were utilized for downstream analyses (27). Cell clustering was performed via the Louvain algorithm implemented in Seurat’s FindClusters function with a resolution parameter of 0.8, resulting in the identification of 18 distinct cell populations. UMAP was used for dimensionality reduction (RunUMAP function), applying the top 30 principal components, with min.dist set to 0.3 and n.neighbors to 30. Cell type annotation was based on canonical marker gene expression, cross-validated with published datasets (9), the CellMarker database (http://biocc.hrbmu.edu.cn/CellMarker/), and established marker genes (**Table 1**). Differentially expressed genes (DEGs) for each cluster were determined and subjected to functional enrichment analysis using the clusterProfiler or msigdbr packages, including KEGG pathway, HALLMARK gene set, and GO Biological Process enrichment. The activity scores (AUC) for the GO:0006954 and GO:0006979 gene sets were computed for each cell and cluster using gene set scoring tools such as AUCell.

**Table 1:** Cell markers.

Cell type	Markers
Fibroblasts	Dpt, Sfrp4, Postn
Schwann cells	Nrgf,Sox10
T cells	Cd3e
Pericytes	Rgs5, Vtn
Activated Neural Stem cells	Gpc3, Myoc
Endothelial cells	Plvap
Smooth Muscle cells	Pln, Mustn1
NK cells	Nkg7
Microglia	Cyb561a3, Siglech, Gapt
Neurons	Slc16a11, Asgr1, Cyp2s1
Neutrophils	Klrc3
CD4+ T cells	G0s2, Napsa
Myeloid cells	Ptprcap
Dendritic cells	Eno3, Slc7a11
Macrophages	Aif1, Cd68, Pf4
M2 Macrophages	Mrc1, Cd163
M1 Macrophages	Il1rn, Cd86, Il1b
Intermediate Macrophages	Cd163, Il1rn
Proliferating Macrophages	Mki67

### 2.2 Chemicals and reagents

Gallic acid (F103701; 99% purity) was acquired from Shanghai Aladdin Biotechnology Co., Ltd. (China). Mecobalamin (lot number: 1703098) was from Eisai Pharmaceutical Co., Ltd. (China). PVDF membranes were acquired from Millipore (Billerica, USA), and RIPA buffer (WB-0071) was acquired from Beijing Dingguo Biological Co., Ltd. (China). SYBR Green Premix qPCR, an RT–PCR Kit, and RNase-Free water (AG11701, AG11602, AG11012) were purchased from Accurate Biotechnology Co., Ltd. (China). IL-8, COX2, TNF-α, IL-6, IL1β, TGF-β ELISA kits (MM-0175R1, MM-70321R1, MM-0180R1, MM-0190R1, MM-0047R1, MM-20594R1 respectively) were obtained from MEIMIAN Industrial Co., Ltd. (China). LPS (L2880, Sigma), GLX351322(a NOX4 inhibitor, APExBIO) were acquired from Guangzhou Yiyou Biological Co., Ltd. (China). The antibodies used were as follows were as follows: PE anti-mouse CD32 Antibody (156404, Biolegend), APC anti-mouse CD206 Antibody (141708, Biolegend), anti-COX2 (WL01750, Wanleibio), anti-iNOS (2982S, CST), anti-NOX4 (ZENBIO,380874), anti-ATF4(ET1612-37, HUABIO), anti-IBA-1(Bimake, A5595), anti-TNF-α(ZENBIO, 346654), anti-Nrf2 (Affinity,BF8017), anti-p-Nrf2 (Affinity,DF7519), anti-IL1β (Wanlei-Bio,WL00891), and anti-β-actin (ZENBIO, 380624). DCFH-DA (D6470) was acquired from Beijing Solarbio Science & Technology Co., Ltd.

### 2.3 Cytotoxicity assay

A CCK-8 assay kit was used to assess cell viability. RAW264.7 cells were seeded at a density of 5×10³ cells/well in a 96-well plate and incubated for 24 hours to allow adhesion. Cells were then treated with various concentrations of LPS (0, 0.25, 0.5, 1, 2.5, 5, 10 µg/mL), GLX351322 (0, 2.5, 5, 10, 25, 50, 100 µM), and GA (0, 2.5, 5, 10, 25, 50, 100, 250, 500, 1000 µM) for 24 hours. After treatment, 10 μl of CCK-8 solution was added to each well and incubated for 2 hours. The absorbance at 450 nm was measured using a microplate reader (BioTek Epoch 2, Vermont, USA).

### 2.4 Cell culture and treatment

Mouse monocyte-macrophage leukaemia cells (RAW264.7 cells) were obtained from Huatuo Biotechnology Co., Ltd. (China). RAW264.7 cells were cultured in DMEM supplemented with 10% fetal bovine serum and 1% penicillin-streptomycin, and incubated in a humidified incubator at 37 °C with 5% CO_2_. RAW264.7 cells were treated with: (1) LPS (1 μg/ml) alone;(2) LPS and GLX351322 (10 μM); (3) gallic acid (GA, 10 μM) alone;(4) LPS and GA. All chemicals were administered simultaneously.

### 2.5 ROS measurement

The ROS were detected using the DCFH-DA fluorescent probe. RAW264.7 cells were seeded in 6-well plates and allocated to experimental groups. After 24 h of treatment, cells were washed three times with PBS and incubated with 1 mL PBS containing 20 μM DCFH-DA at 37 °C for 30 min in the dark. After removing the dye and washing once with PBS, ROS levels were visualized by fluorescence microscopy (excitation/emission ~488/525 nm).

For quantitative ROS assessment, cells were trypsinized, transferred to centrifuge tubes, and washed three times with PBS. The cell pellet was resuspended and incubated in the dark with 1 mL PBS containing 20 μM DCFH-DA per tube at 37 °C for 30 min, with gentle agitation every 3-5 min. After incubation, the dye was aspirated and cells were washed three times with PBS. Fluorescence intensity, indicative of intracellular ROS, was measured by flow cytometry (Beckman Coulter, Inc., USA) (28).

### 2.6 Flow cytometric analysis of macrophage M1/M2 polarization

Cells were plated in 6-well dishes at 1×10^6^ cells/well and incubated as described above for 24 hours. Cells were digested with trypsin, washed, and resuspended in cold PBS at 1×10^6^ cells/mL. Membrane protein CD32 was detected by direct staining: cells were fixed, permeabilized, and incubated with PE-conjugated mouse CD32 antibody in the dark for 30 minutes (29). For MRC1, cells were fixed, blocked, incubated with MRC1 polyclonal antibody, and then with DyLight 638-conjugated goat anti-rabbit IgG for 30 minutes in the dark. Cells were washed, resuspended in 500 μL PBS, and 10^5^ cells per sample were analysed using a flow cytometer (CytoFLEX, Beckman Coulter, USA) with FlowJo software.

### 2.7 Animals

The Ethics Committee of Animal Research, Jinan University approved all animal procedures. The research adhered to the NIH(Institute of Laboratory Animal Resources, U.S.) Guide for the Care and Use of Laboratory Animals, and the animals were housed in a standard environment with a 12h light/dark cycle, a temperature of 24 ± 1 °C, and a humidity of 55 ± 5%. Thirty-two male SD rats (180–220 g, 6–8 week old) were provided by Guangdong Weitong Lihua Co., Ltd(China). The rats were placed in a standard SPF-ventilated laboratory with five rats per cage, provided with sufficient food and water, and bedding was changed regularly. An application for ethical approval was submitted on 8 November 2022 (No. 20221114-02) and ethical approval was granted on the same date (No. IACUC-20221114-02).

### 2.8 CCI model

Rats were anaesthetised with an intraperitoneal injection of 2% sodium pentobarbital at a dose of 0.2 ml per 100 g body weight. After anesthesia induction, the fur over the posterior thigh (covering at least a 6 cm area around the surgical site) was shaved and the skin disinfected. The muscles were carefully separated to reveal the sciatic nerve proximal to its bifurcation. Using 4–0 silk sutures, the sciatic nerve was ligated at four distinct sites, each ligature spaced 1 mm apart (27, 30–32). This resulted in mild indentation of the epineurium and slight contraction of the right lower limb muscles. After completion of the ligation, the incision was closed with sutures, and penicillin was administered to prevent postoperative infection. The rats were randomly divided into four groups (n=8/group):(1) Sham group (sham operation: skin incision only, then sutured; 0.9% NaCl 2 ml/kg/day, twice daily, intragastric administration (i.g.));(2) CCI group (CCI operation; 0.9% NaCl 2 ml/kg/day,twice daily, i.g.);(3) Mebl group (CCI operation; mecobalamin 10 mg/kg twice daily, i.g.) (33);(4) GA group (CCI operation; gallic acid 50 mg/kg twice daily, i.g.) (34). The treatment lasted for 21 days.

### 2.9 Behavioral tests

Thermal hyperalgesia was evaluated using a hot-plate apparatus (YLS-6B, Jinan Yiyan Technology Development Co., Ltd., China). Thermal sensitivity was assessed by briefly placing the lateral plantar surface of the right hind paw on a hot plate maintained at 50 °C, and the time (paw−withdrawal latency, PWT) it took for the rat to raise its right foot was recorded (33).A predefined cut-off latency(40s) was used in the hot-plate test to prevent potential tissue injury.

### 2.10 Hematoxylin–eosin staining

Tissues (liver, kidney, and sciatic nerve) were fixed in 4% paraformaldehyde, processed for paraffin embedding, and sectioned. Sections were deparaffinized in xylene, rehydrated through a graded ethanol series, rinsed in distilled water, and stained with hematoxylin for 3–5 min, followed by differentiation and bluing according to standard procedures. After rinsing, sections were dehydrated in 85% and 95% ethanol for 5 min each, counterstained with eosin for 5 min, further dehydrated in absolute ethanol, cleared in xylene, and mounted with neutral gum. Histological evaluation was performed using a Leica light microscope, and images were captured for analysis (10× for general morphology and 40× for higher-resolution evaluation).

### 2.11 ELISA

For quantification of inflammatory mediators in the right sciatic nerve, tissues were homogenized in saline and centrifuged at 3,000 rpm for 10 min. The supernatants were collected and analyzed according to the manufacturer’s instructions. Optical density was measured using a microplate reader (BioTek Instruments, Inc.).

### 2.12 Immunofluorescent staining

For immunohistochemistry, paraffin-embedded sections were dewaxed, rehydrated, and subjected to antigen retrieval. Endogenous peroxidase activity was blocked by incubating the sections in a peroxidase-blocking solution (protected from light) for 25 min (35). Sections were then incubated with goat anti-rabbit serum for 30 min to block nonspecific binding, followed by overnight incubation at 4 °C with primary antibodies. After incubation with the appropriate secondary antibodies and streptavidin, immunoreactivity was visualized using a DAB substrate kit according to the manufacturer’s instructions. For quantitative analysis, images from three randomly selected fields per specimen were acquired using a Leica light microscope. Two blinded observers independently quantified staining using ImageJ software (10× for general morphology and 40× for higher-resolution evaluation).

Paraffin sections were dewaxed and rehydrated to water, subjected to antigen retrieval, circled with a hydrophobic barrier pen, and blocked with 3% BSA for 30 min. Sections were then incubated overnight at 4 °C with primary antibody mixtures (CD32 or MRC1 together with IBA-1), followed by incubation with the corresponding secondary antibodies for 50 min in the dark. Nuclei were counterstained with DAPI for 10 min at room temperature. Finally, an anti-fade reagent was applied, and sections were mounted with an anti-fade mounting medium. Images were acquired under the following settings: DAPI (excitation 330–380 nm, emission 420 nm); FITC (excitation 465–495 nm, emission 515–555 nm; green); and Cy3 (excitation 510–560 nm, emission 590 nm; red). Images were captured at 10× for general morphology and 40× for higher-resolution evaluation.

### 2.13 Western blot

Sciatic nerve tissues and cells were lysed in lysis buffer containing protease and phosphatase inhibitor cocktails for 30 min, followed by centrifugation at 12,000 rpm for 10 min at 4 °C (11, 36, 37). The supernatants were collected, and total protein concentrations were determined using a BCA protein assay kit. Equal amounts of protein (5 µg) were mixed with loading buffer, denatured at 95 °C for 5 min, and separated on 10% Bis–Tris SDS–PAGE gels (1.0 mm, 10-well). Proteins were then transferred to PVDF membranes, which were blocked and incubated overnight at 4 °C with primary antibodies (37, 38). After washing, membranes were incubated with species-appropriate HRP-conjugated secondary antibodies for 1 h, developed using a luminol-based chemiluminescent substrate, and imaged with a ChemiDoc MP imaging system. Band intensities were quantified by densitometric analysis using ImageJ.

### 2.14 PCR analysis

RNA was extracted using RNAiso Plus and reverse-transcribed into cDNA using an RT–qPCR kit. qPCR was performed with SYBR Green PCR Mix and gene-specific primers (**Table 2**) on a Bio-Rad real-time PCR system (Bio-Rad Corporation, USA). After normalization to β-actin, relative mRNA expression levels were calculated using the 2^−ΔΔCt^ method (11, 15, 26, 39).

**Table 2:** Primer sequences.

Gene	Forward primer	Reverse primer
β-Actin	CTACCTCATGAAGATCCTCACCGA	TTCTCCTTAATGTCACGCACGATT
IL1β	GAAATGCCACCTTTTGACAGTG	TGGA TGCTCTCATCAGGACAG
iNOS	GTTCTCAGCCCAACAATACAAGA	GTGGACGGGTCGATGTCAC
CD32	TGGACAGCCGTGCTAAATCTT	GGTCCCTTCGCATGTCAGTG
CD206	CTCTGTTCAGCTATTGGACGC	CGGAATTTCTGGGATTCAGCTTC
TNF-α	CTGAACTTCGGGGTGATCGG	GGCTTGTCACTCGAATTTTGAGA
NOX4	TTTGCCTGGAAGAACCCAAGT	CAGGTTTGTTGCTCCTGATGC
MDA	CAACTCTTTCCTCTGCGTGC	GCATTGGTGGCTTCCTGACT

### 2.15 Statistical analysis

All data were analyzed using GraphPad Prism 9 and are presented as the mean ± standard deviation (SD). Normality was assessed using the Shapiro–Wilk test. For datasets that met the normality assumption, parametric tests were applied; otherwise, nonparametric tests were used. Behavioral tests were analyzed using two-way ANOVA with repeated measures on time, with group as the between-subject factor and time as the within-subject factor, followed by Tukey’s multiple comparisons test.

Methods for sequencing data analysis have been described previously. Data from other experiments, including ELISA, qPCR, Western blotting, and immunohistochemistry, were analyzed using one-way ANOVA followed by Tukey’s multiple-comparisons *post hoc* test. Statistical significance was set at *p < 0.05*.

## 3 Results

### 3.1 Classification of major cell types within the sciatic nerve after peripheral nerve injury

**Figure 1:**
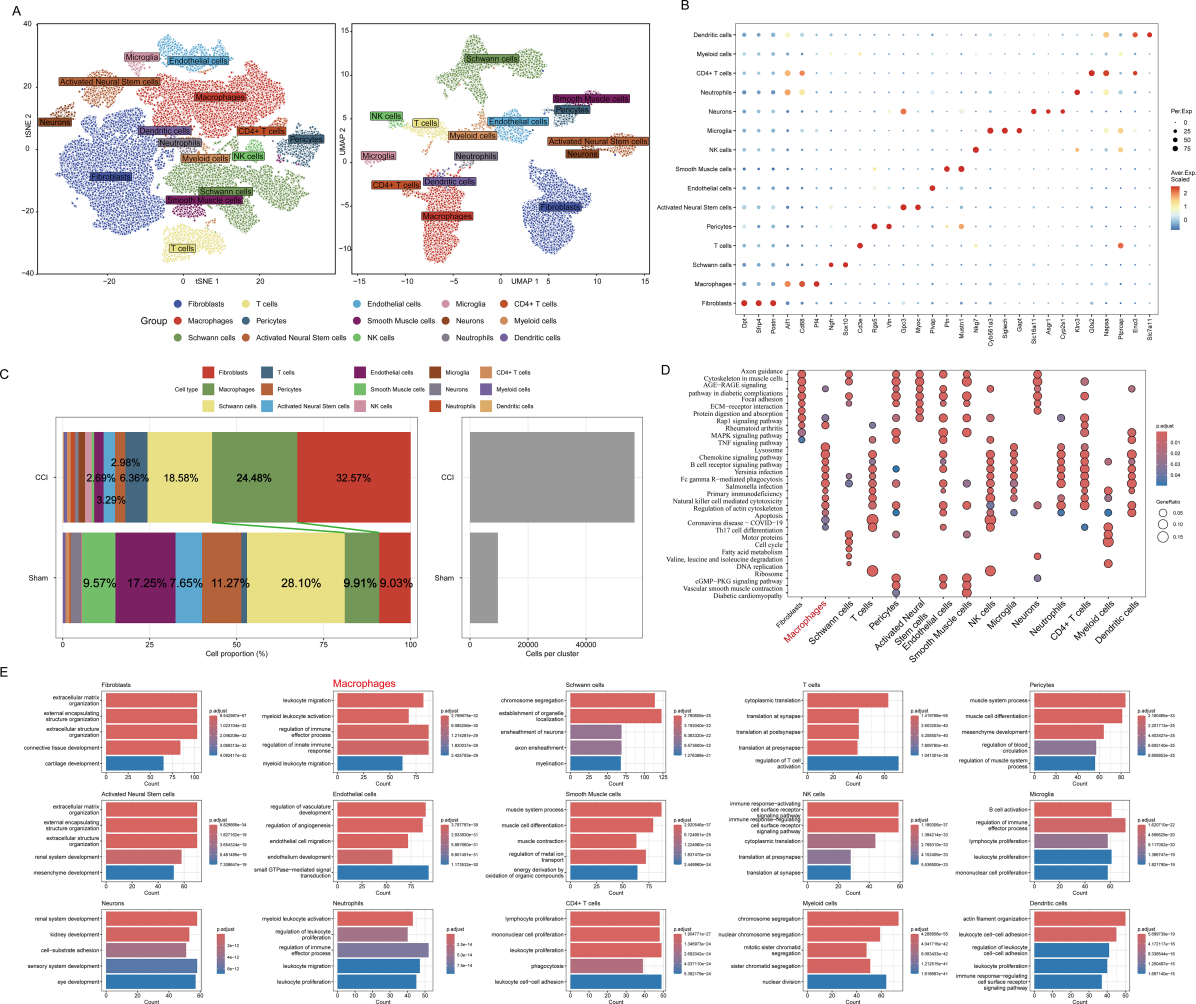
Changes in cell proportion and differential gene expression analysis after injury. **(A)** Annotated UMAP and tSNE plots. **(B)** Heatmap of cell type markers. **(C)** Proportions of various types of cells. **(D)** KEGG results. **(E)** GO results.

To investigate changes in cell-type composition after nerve injury, we performed scRNA-seq analysis. Unsupervised clustering revealed 28 clusters based on known markers (**Figure 1B**), and the differentially expressed genes could be classified into multiple cell types (**Figure 1A**), including fibroblasts (Dpt, Sfrp4, Postn), Schwann cells (Nrgf, Sox10), T cells (Cd3e), pericytes (Rgs5, Vtn), activated neural stem cells (Gpc3, Myoc), endothelial cells (Plvap), smooth muscle cells (Pln, Mustn1), NK cells (Nkg7), microglia (Cyb561a3, Siglech, Gapt), neurons (Slc16a11, Asgr1, Cyp2s1), neutrophils (Klrc3), CD4^+^ T cells (G0s2, Napsa), myeloid cells (Ptprcap), dendritic cells (Eno3, Slc7a11), and macrophages (Aif1, Cd68, Pf4). The proportions of different cell types are shown in **Figure 1C** (**Table 3**). The number of macrophages increased from 952 cells (9.91%) in the naïve sciatic nerve to 13,875 cells (24.49%) after injury. Similarly, the number of T cells increased from 155 cells (1.61%) to 3,606 cells (6.36%) in the two groups (**Figure 1C**). KEGG enrichment analysis revealed that “chemokine signaling pathway” and “cytokine–cytokine receptor interaction” (**Supplementary Figure 1**) were among the top five enriched pathways. GO analysis further indicated that “regulation of immune effector processes” and “regulation of the innate immune response” were involved in sciatica pathogenesis (**Figure 1E**).

**Table 3:** The proportions and cell number of various types of cells.

Cell type	Sham		CCI	
Fibroblasts	9.04%	868	32.58%	18460
Macrophages	9.91%	952	24.49%	13875
Schwann cells	28.10%	2699	18.58%	10530
T cells	1.61%	155	6.36%	3606
Pericytes	11.28%	1083	2.98%	1688
Activated Neural Stem cells	7.65%	735	3.29%	1865
Endothelial cells	17.25%	1657	2.70%	1529
Smooth Muscle cells	9.58%	920	0.68%	386
NK cells	0.26%	24	1.95%	1104
Microglia	0.08%	8	1.84%	1045
Neurons	2.93%	281	1.05%	595
Neutrophils	0.36%	35	1.23%	695
CD4+ T cells	1.12%	108	1.04%	591
Myeloid cells	0.79%	76	0.94%	534
Dendritic cells	0.02%	2	0.29%	164

### 3.2 Increased inflammatory response and oxidative stress after CCI

**Figure 2:**
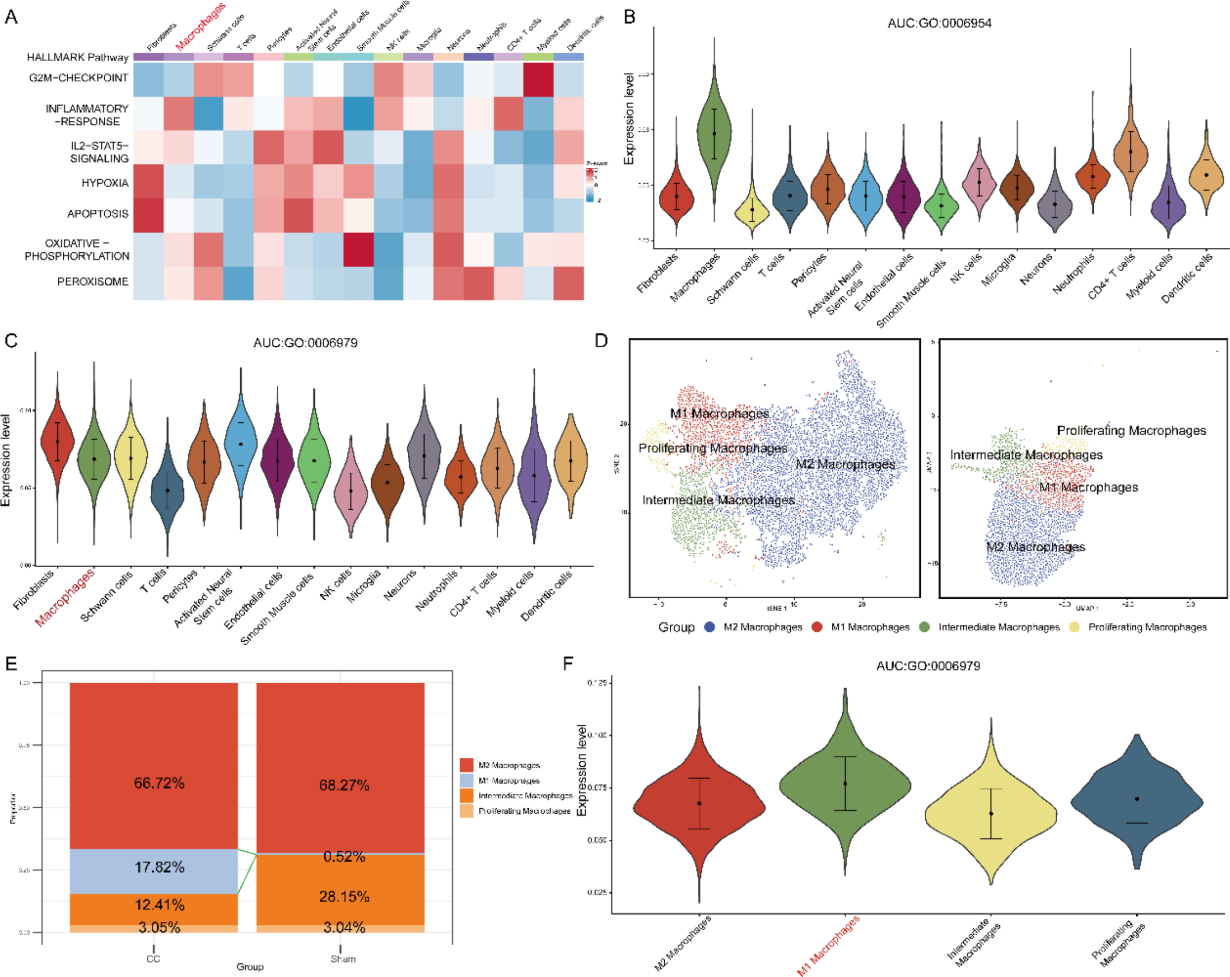
Analysis of macrophage subtypes, GSEA results, and AUCs. **(A)** Hallmark GSEA. **(B)** AUC for the GO:0006954 gene set. **(C)** AUC for the GO:0006979 gene set. **(D)** UMAP and t-SNE plots of macrophage subtypes. **(E)** Proportions of M1 and M2 macrophages. **(F)** AUC for the GO:0006979 gene set in macrophages.

Hallmark GSEA indicated that the “HALLMARK_INFLAMMATORY_RESPONSE” gene set was significantly enriched, suggesting its potential role in pain persistence (**Figure 2A**). We then calculated AUC-based module scores for the GO:0006954 (inflammatory response; **Figure 2B**) and GO:0006979 (response to oxidative stress; **Figure 2C**) gene sets, which revealed increased inflammatory response and oxidative stress scores in macrophages after CCI. Macrophage subtype analysis further revealed a marked increase in the proportion of M1 macrophages after CCI (from 0.52% to 17.82%; **Figures 2D, E**). In addition, M1 macrophages from CCI-treated samples presented increased oxidative stress scores (**Figure 2F**).

### 3.3 Cytotoxic effects of LPS, GLX351322, and GA on RAW264.7 cells

**Figure 3:**
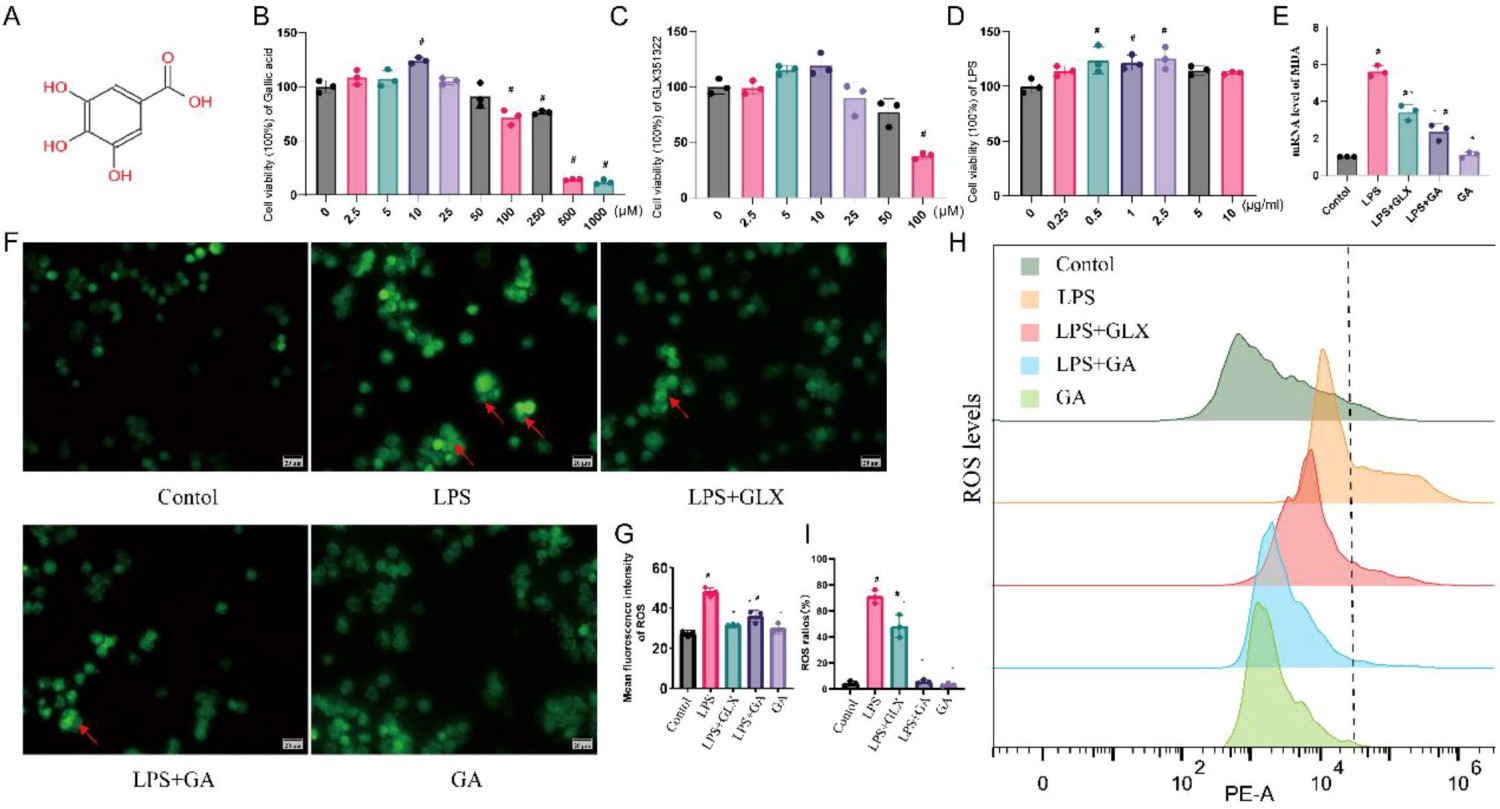
GA reduced ROS generation in RAW264.7 cells after LPS treatment. **(A)** The chemical structure of GA. **(B–D)** The cytotoxic effects of LPS, GLX351322, and GA on RAW264.7 cells. **(E)** mRNA level of MDA. **(F, G)** ROS fluorescence. **(H, I)** Fluorescence intensity by flow cytometry. ^#^compared with the control group. *compared with the LPS group. *p* < 0.05.

GA, GLX351322, and LPS were not significantly cytotoxic at the concentrations used for the subsequent functional experiments (GA ≤ 10 μM; GLX351322 ≤ 10 μM; LPS = 1 μg/mL; **Figures 3B–D**). By contrast, high concentrations of GA (≥ 250 μM) significantly reduced cell viability, and GLX351322 decreased cell viability when its concentration was ≥50 μM.

### 3.4 GA attenuated ROS levels in RAW264.7 cells after LPS treatment

LPS significantly increased the intracellular ROS and MDA levels in RAW264.7 cells (**Figure 3E**, *p* < 0.05). After treatment with GA and GLX351322, the levels of MDA (**Figure 3E**, *p* < 0.05) and ROS (**Figures 3F–I**, *p* < 0.05) decreased. Individually, GA did not increase the levels of ROS or MDA but did reduce their levels after LPS stimulation to normal levels (*p* < 0.05). In addition, GLX351322 did not decrease ROS and MDA levels to the normal levels.

### 3.5 GA promoted the transition of RAW264.7 cells from M1 polarization to M2 polarization after LPS stimulation

**Figure 4:**
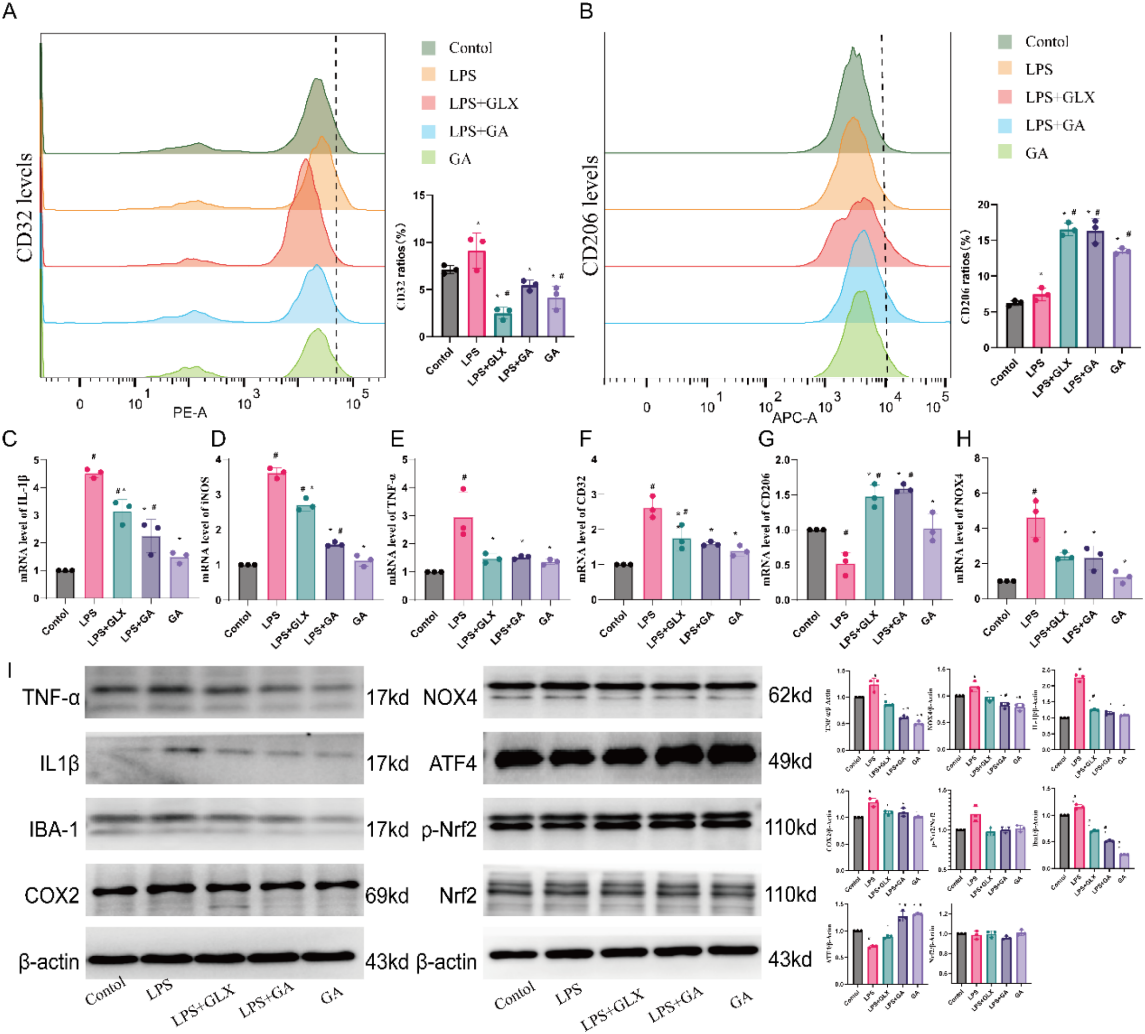
GA attenuated the LPS-induced M1 polarization of RAW264.7 cells. **(A)** Fluorescence intensity of M1-polarized macrophages (CD32) measured by flow cytometry. **(B)** Fluorescence intensity of M2-polarized macrophages (CD206) measured by flow cytometry. **(C–F)** Relative expression levels of M1 markers (IL-1β, TNF-α, iNOS, and CD32). **(G)** Relative expression levels of M2 markers (CD206). **(H)** mRNA level of NOX4. **(I)** Western blots (IL-1β, TNF-α, IBA-1, COX2, NOX4, Nrf2, p-Nrf2). ^#^compared with the control group. *compared with the LPS group. *p* < 0.05.

As shown in **Figure 4** A and B, LPS stimulation increased the proportions of CD32 and CD206 in RAW264.7 cells, whereas GLX351322 and GA clearly reduced the CD32 level and increased the CD206 level (**Figures 4A, B**, *p* < 0.05). GA decreased the CD32 level to normal (**Figure 4A**, *p* < 0.05). GA increased the CD206 level but did not increase the CD32 level. The mRNA levels of IL-1β, iNOS, TNF-α, CD32, and CD206 are shown in **Figures 4C–G**. The mRNA expression levels of the M1 polarization markers IL-1β, iNOS, TNF-α, and CD32 increased after LPS stimulation (**Figures 4C–G**, *p* < 0.05), whereas that of CD206 remained unchanged. However, GA decreased the levels of IL-1β, iNOS, TNF-α, and CD32, whereas increased the CD206 level (*p* < 0.05).

### 3.6 GA attenuated the LPS-induced M1 polarization of RAW264.7 cells through NOX4-mediated oxidative stress

After LPS treatment, the expression levels of NOX4, TNF-α, IBA-1, IL-1β, COX2, and iNOS in RAW264.7 cells significantly increased (**Figure 4I**, *p* < 0.05), and the level of NOX4 mRNA increased (**Figure 4H**, *p*< 0.05), which was consistent with the mRNA results. The levels of ATF4 and p-Nrf2 decreased (**Figure 4I**, *p* < 0.05), and the level of Nrf2 did not vary. After treatment with GA, the levels of ATF4 and p-Nrf2 increased, whereas those of NOX4, TNF-α, IBA-1, IL-1β, COX2, and iNOS decreased (**Figure 4I**, *p* < 0.05).

### 3.7 GA alleviated thermal hyperalgesia in CCI rats

**Figure 5:**
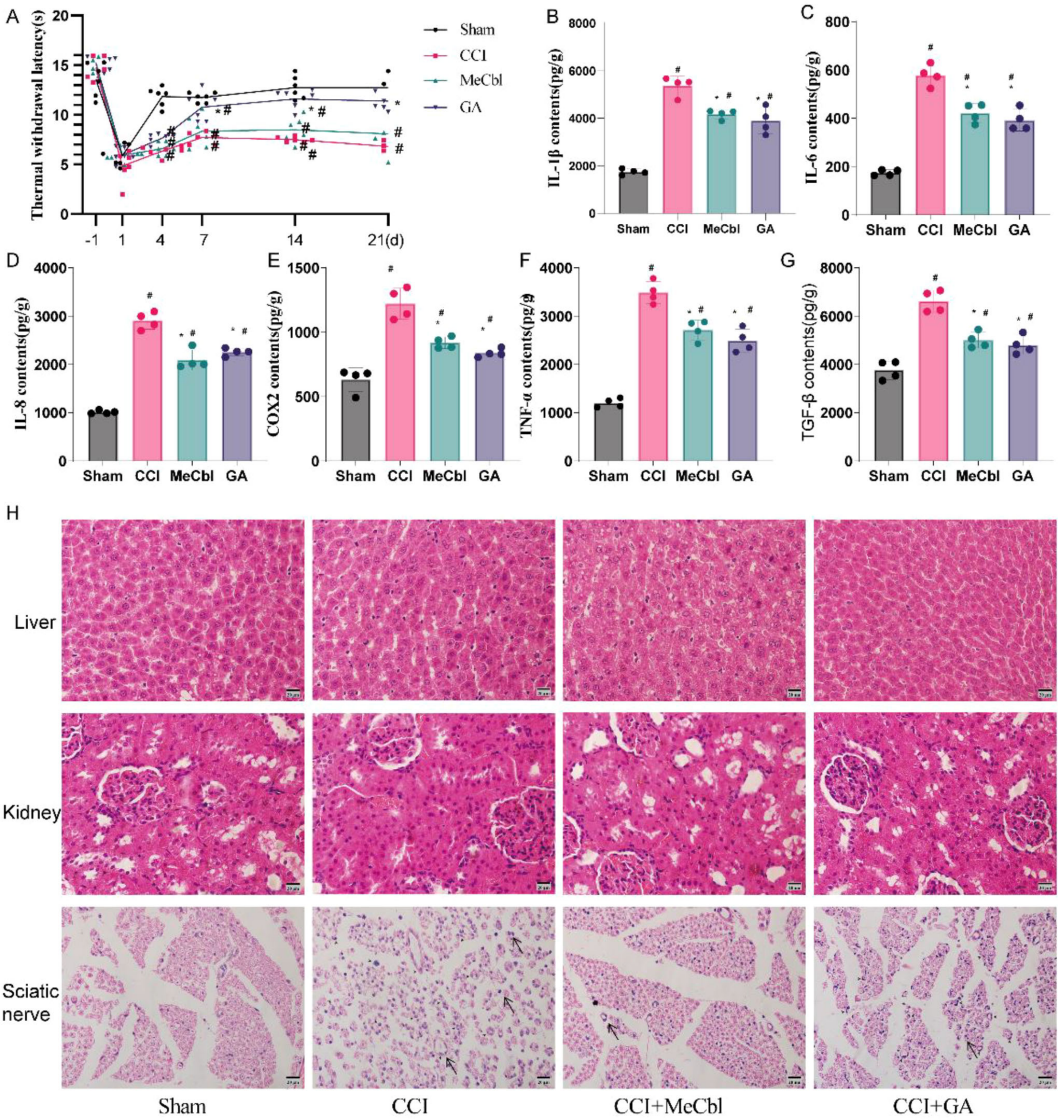
GA alleviated pain and reduced the inflammatory response. **(A)** Behavioral tests of hot plates. **(B–G)** ELISA-based determination of the levels of IL-8, COX2, TNF-α, IL-6, IL-1β, and TGF-β in the sciatic nerve. **(H)** H&E staining of the right sciatic nerve, liver, and kidney. ^#^Compared with the sham operation group; *compared with the CCI group; *p* < 0.05.

On the first postoperative day, all the rats developed varying degrees of thermal hyperalgesia. In the sham-operated group, thermal hyperalgesia gradually returned to baseline levels from Days 4 to 21. Compared with those in the Sham group, rats in the CCI model group showed significant thermal hyperalgesia from Days 4 to 21 (**Figure 5A**, *p* < 0.05). Notably, compared with the CCI group, the GA and mecobalamin exhibited significantly attenuated CCI-induced thermal hyperalgesia and increased thermal withdrawal latency, but these values did not necessarily return completely to baseline (**Figure 5A**, *p* < 0.05).

### 3.8 Effect of GA on the levels of inflammatory factors in the sciatic nerve of CCI rats

ELISA results (**Figure 5B–G**) revealed that on postoperative Day 21, the levels of the inflammatory factors IL-8, COX-2, TNF-α, TGF-β, IL-6, and IL-1β in the sciatic nerve in the CCI group were significantly greater than those in the sham rats (*p* < 0.05). After treatment with mecobalamin and GA, the levels of IL-8, COX-2, TNF-α, TGF-β, IL-6, and IL-1β were lower than those in the CCI group (*p* < 0.05), whereas the levels in the GA group did not decrease to normal.

### 3.9 GA improves histopathological changes in the sciatic nerve

The liver and kidney tissues of each group all showed normal histological morphology (**Figure 5H**), indicating that CCI surgery, mecobalamin, and GA administration did not cause damage to the aforementioned organs.

H&E staining of the sciatic nerve revealed structural integrity and no inflammatory cell infiltration in the sham group, whereas in the CCI group, the sciatic nerve structure was disrupted, characterized by disorganized nerve fibers, extensive Schwann cell proliferation, and severe inflammatory infiltration (**Figure 5H**). Mecobalamin and GA exerted significant therapeutic effects on damaged sciatic nerves, including alleviating inflammatory infiltration and restoring the arrangement of nerve fibers.

### 3.10 Coexpression analysis of IBA-1/CD32 and IBA-1/CD206 in the sciatic nerve

**Figure 6:**
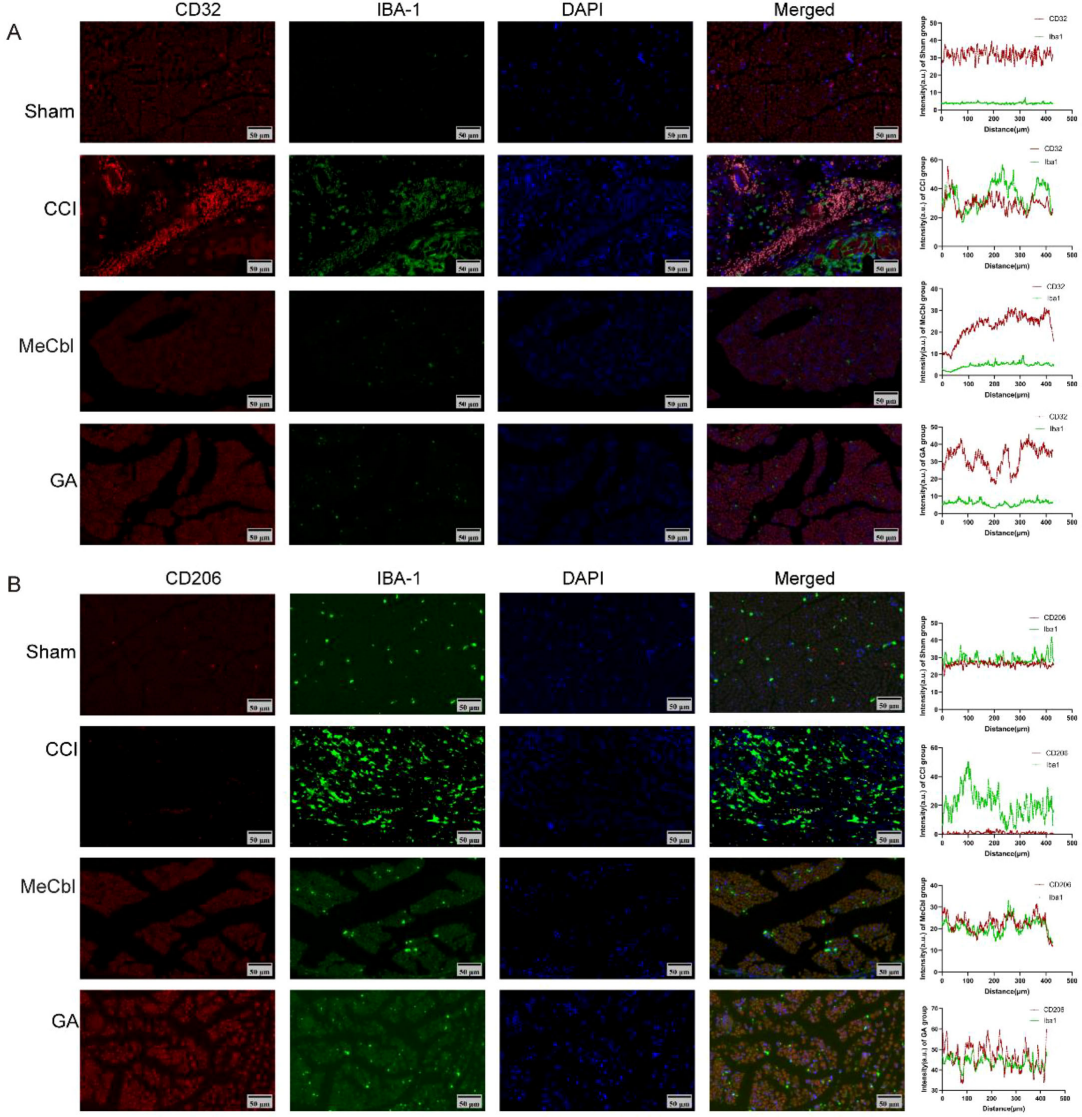
GA affected the activation of M1/M2 macrophages. **(A, B)** Coexpression analysis of IBA-1/CD32 and IBA-1/CD206. ^#^Compared with the sham operation group; *compared with the CCI group; *p* < 0.05.

The CCI operation increased the expression levels of IBA-1 and CD32 and decreased the expression level of CD206, whereas GA and mecobalamin reversed these trends. In addition, we assessed the colocalization of IBA-1/CD32 and IBA-1/CD206 using ImageJ (Plot Profile, JACOP, Pearson’s correlation coefficient), which revealed that CCI increased the coexpression of IBA-1/CD32 and decreased the coexpression of IBA-1/CD206 (**Figures 6A, B**). After treatment with GA, the coexpression of IBA-1/CD32 decreased, whereas that of IBA-1/CD206 increased.

### 3.11 Effects of GA on NOX4-mediated oxidative stress

**Figure 7:**
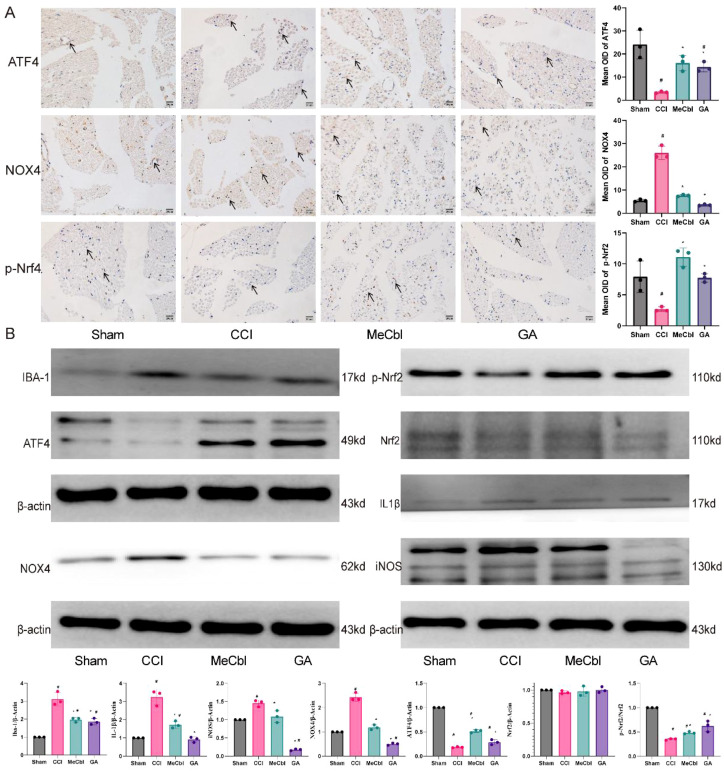
GA reduced NOX4-mediated oxidative stress. **(A)** Immunohistochemistry of NOX4, ATF4, and p-Nrf2. **(B)** Western blots of NOX4, ATF4, p-Nrf2, Nrf2, IL-1β, iNOS, and IBA-1 expression. ^#^Compared with the sham operation group; *compared with the CCI group; *p* < 0.05.

Immunohistochemical staining for NOX4, ATF4, and p-Nrf2 (**Figure 7A**) revealed that CCI increased the level of NOX4 and decreased the levels of ATF4 and p-Nrf2. By contrast, GA and mecobalamin decreased the expression level of NOX4 and increased the expression levels of ATF4 and p-Nrf2 (**Figure 7A**, *p* < 0.05).

The levels of NOX4, ATF4, p-Nrf2, Nrf2, IL-1β, iNOS, and IBA-1 were detected by western blotting. CCI increased the levels of IBA-1, NOX4, IL-1β, and iNOS and decreased the levels of ATF4 and p-Nrf2 but did not affect the level of Nrf2. By contrast, GA and mecobalamin decreased the expression levels of IBA-1, NOX4, IL-1β, or iNOS and increased the expression levels of ATF4 and p-Nrf2 (**Figure 7B**, *p* < 0.05).

## 4 Discussion

In this study, the experimental results suggest that GA may alleviate sciatica-like symptoms in a rat CCI model. The study findings are as follows: (1) bioinformatics analysis revealed that “inflammatory response” and “response to oxidative stress” contribute to the development of sciatica; (2) GA attenuated ROS levels in macrophages after LPS stimulation and the inflammatory response; (3) GA attenuated LPS-induced M1 polarization by inhibiting NOX4-mediated oxidative stress; and (4) GA alleviated thermal hyperalgesia in CCI rats by inhibiting inflammatory factors and promoting neural repair.

The CCI model is a classic animal model for neuropathic pain (9, 31) and is characterized by persistent hyperalgesia and spontaneous pain after nerve compression (9, 11, 15, 27). The transient thermal hyperalgesia observed in the sham group during the first 4 days likely reflects the effects of surgical tissue injury (incisional trauma) and the associated acute postoperative inflammation (40–42), rather than neuropathic pain resulting from nerve ligation. GA significantly increased the paw withdrawal latency after CCI surgery, indicating that it can relieve pain.Previously studies have supported the analgesic potential of gallic acid and its derivatives. Gallic acid ethyl ester has been reported to exert antinociceptive effects through mechanisms involving K^+^ channels and Gi/o proteins (43). Gallic acid also acts as a TRPA1 antagonist and shows antinociceptive and antiedematogenic effects in mice (44). Moreover, gallic acid improved pain-related outcomes in a rat model of transient global ischemia/reperfusion injury (45). Together, these findings further support the pain-modulating effects of gallic acid observed in the present study (46). Mecobalamin is widely used clinically for peripheral nerve injury and neuropathic symptoms (47) and is commonly used as a reference drug in experimental neuropathic pain studies. Following peripheral nerve injury, a rapid proinflammatory response is essential for clearing tissue debris and promoting effective nerve regeneration (48). If inflammation persists and is not promptly and effectively suppressed, functional recovery is compromised, indicating that suppression of the inflammatory response contributes to functional recovery from peripheral nerve injury. GA promoted nerve repair and attenuated neuroinflammation, significantly reducing the levels of IL-8, COX-2, TNF-α, TGF-β, IL-6, and IL-1β. Analysis of macrophage subtypes revealed that CCI increased the proportion of proinflammatory (M1) macrophages and that LPS further increased M1 polarization. In addition, a coexpression analysis of IBA-1/CD32 and IBA-1/CD206 in the sciatic nerve revealed that CCI increased the proportion of M1 macrophages, which was consistent with the results of the bioinformatics analysis and *in vitro* experiments. Treatment with GA reduced the proportion of M1 macrophages and increased the proportion of M2 macrophages both in CCI rats and *in vitro*. These results indicate that the transition of macrophages from the proinflammatory M1 phenotype to the reparative M2 phenotype significantly reduces inflammatory damage and promotes nerve repair.

ROS induce M1 polarization in macrophages (49). In this study, the AUC results for the GO:0006954 and GO:0006979 gene sets indicated higher levels of inflammatory response and oxidative stress in macrophages after CCI and higher levels of oxidative stress in M1 macrophages. The elimination of ROS inhibits macrophage M1 polarization more effectively (50). Ultrasmall iron–gallic acid coordination polymer nanoparticles effectively alleviated oxidative stress and ameliorated inflammation by modulating microglial polarization toward an anti-inflammatory phenotype (51). In this study, GA reduced the level of M1 polarization in RAW264.7 cells after LPS stimulation by decreasing the ROS level.

The NADPH oxidase family should be discussed, as it is the only known enzyme family specifically dedicated to ROS production (52). NOX4 is an NADPH oxidase known to produce ROS and might have a regulatory function during oxidative stress (53). ATF4 is a key transcription factor downstream of PERK/eIF2α signaling. NOX4-derived ROS may contribute to the activation of ATF4, whereas Nrf2 activation can counteract ROS accumulation and potentially modulate ATF4-related stress signaling (54). Isoquercetin promoted redox homeostasis by inhibiting NOX4 activity and activating Nrf2-mediated antioxidant responses to relieve osteoarthritis (55). ATF4 knockdown increased the expression of inflammatory cytokines (56). In this study, LPS and CCI increased the levels of NOX4, ROS, and inflammatory cytokines and decreased the levels of p-Nrf2 and ATF4. By contrast, GA treatment increased the levels of p-Nrf2 and ATF4 and decreased the levels of NOX4, ROS, and inflammatory cytokines. GLX351322 was included as a positive comparator to validate the involvement of oxidative stress in the inflammatory response. These findings indicate that GA may inhibit NOX4 activity and activate Nrf2 and ATF4 pathways, breaking the positive feedback loop between oxidative stress and inflammation and thereby alleviating nerve damage.

As a widely available, cost-effective natural polyphenolic compound, GA demonstrates favorable safety and multi-target properties. In the present study, *in vivo* and *in vitro* experiments revealed that GA caused no detectable toxicity to major organs, such as the liver and kidneys, supporting its promising translational potential. Nevertheless, several limitations remain. First, the efficacy and safety of GA have not yet been validated in higher-order animal models or clinical samples. Second, only a single dose of GA was tested in the pharmacological experiments, precluding the assessment of dose–response relationships and optimal dosing. Moreover, the hot-plate test was used as a primary readout of thermal nociception and central pain-related responses following CCI, but it does not comprehensively assess other sensory modalities, such as mechanical allodynia.

## 5 Conclusion

GA relieved sciatica-like symptoms in a rat CCI model, possibly through promoting the transition of macrophages from the M1 to the M2 phenotype by modulating NOX4-mediated oxidative stress.

## Data Availability

The data used to support the findings of this study are available from the corresponding authors (dizhang0915@jnu.edu.cn and tguo428@jnu.edu.cn).
