# Supplementary material for: Gallic acid exerts therapeutic effects on sciatica by reducing inflammatory responses through the regulation of NOX4-mediated oxidative stress
Source: Front Immunol. 2026 May 13;17:1748652. doi: 10.3389/fimmu.2026.1748652 (PMC13212122; doi:10.3389/fimmu.2026.1748652)
Supplement: Supplementary file 1 [file Presentation1.pptx]

## Slide 1
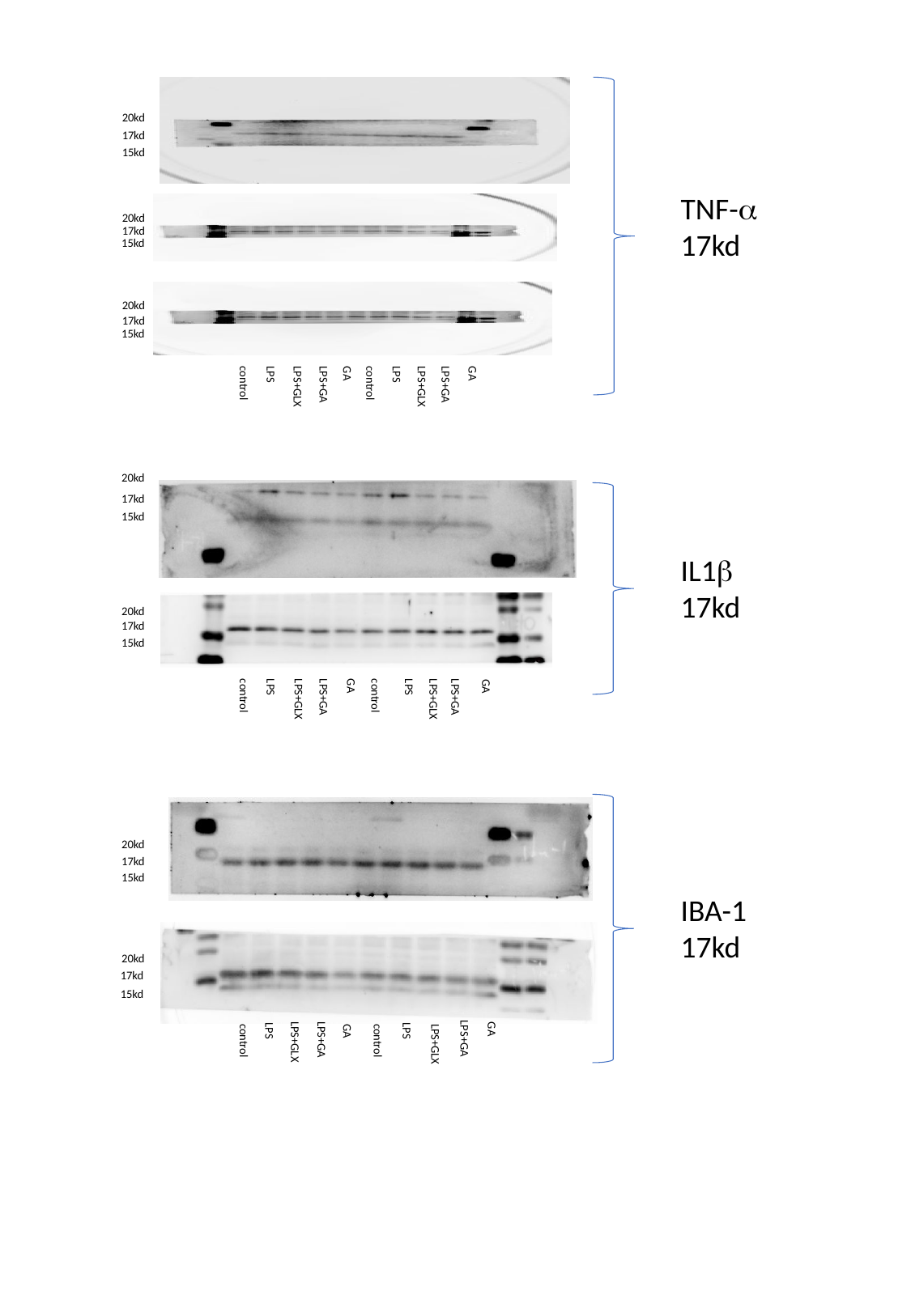

20kd
17kd
15kd
TNF-α
17kd
20kd
17kd
15kd
20kd
17kd
15kd
control
LPS
LPS+GLX
LPS+GA
GA
control
LPS
LPS+GLX
LPS+GA
GA
20kd
17kd
15kd
IL1β
17kd
20kd
17kd
15kd
control
LPS
LPS+GLX
LPS+GA
GA
control
LPS
LPS+GLX
LPS+GA
GA
20kd
17kd
15kd
IBA-1
17kd
20kd
17kd
15kd
LPS+GA
LPS+GLX
LPS+GA
GA
LPS
LPS
control
GA
control
LPS+GLX

## Slide 2
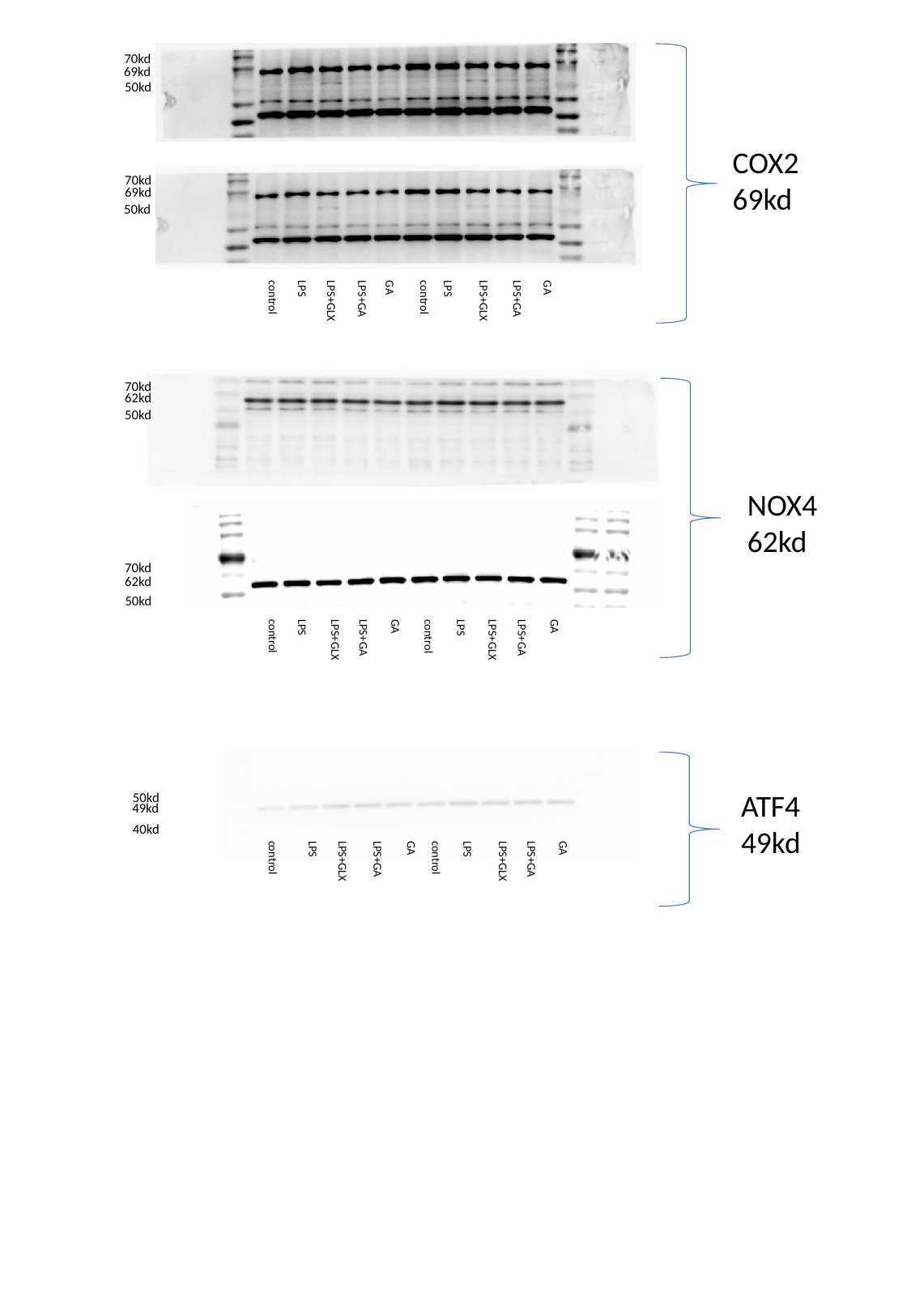

70kd
69kd
50kd
COX2
69kd
70kd
69kd
50kd
control
LPS
LPS+GLX
LPS+GA
GA
control
LPS
LPS+GLX
LPS+GA
GA
70kd
62kd
50kd
NOX4
62kd
70kd
62kd
50kd
control
LPS
LPS+GLX
LPS+GA
GA
control
LPS
LPS+GLX
LPS+GA
GA
ATF4
49kd
50kd
49kd
40kd
control
LPS
LPS+GLX
LPS+GA
GA
control
LPS
LPS+GLX
LPS+GA
GA

## Slide 3
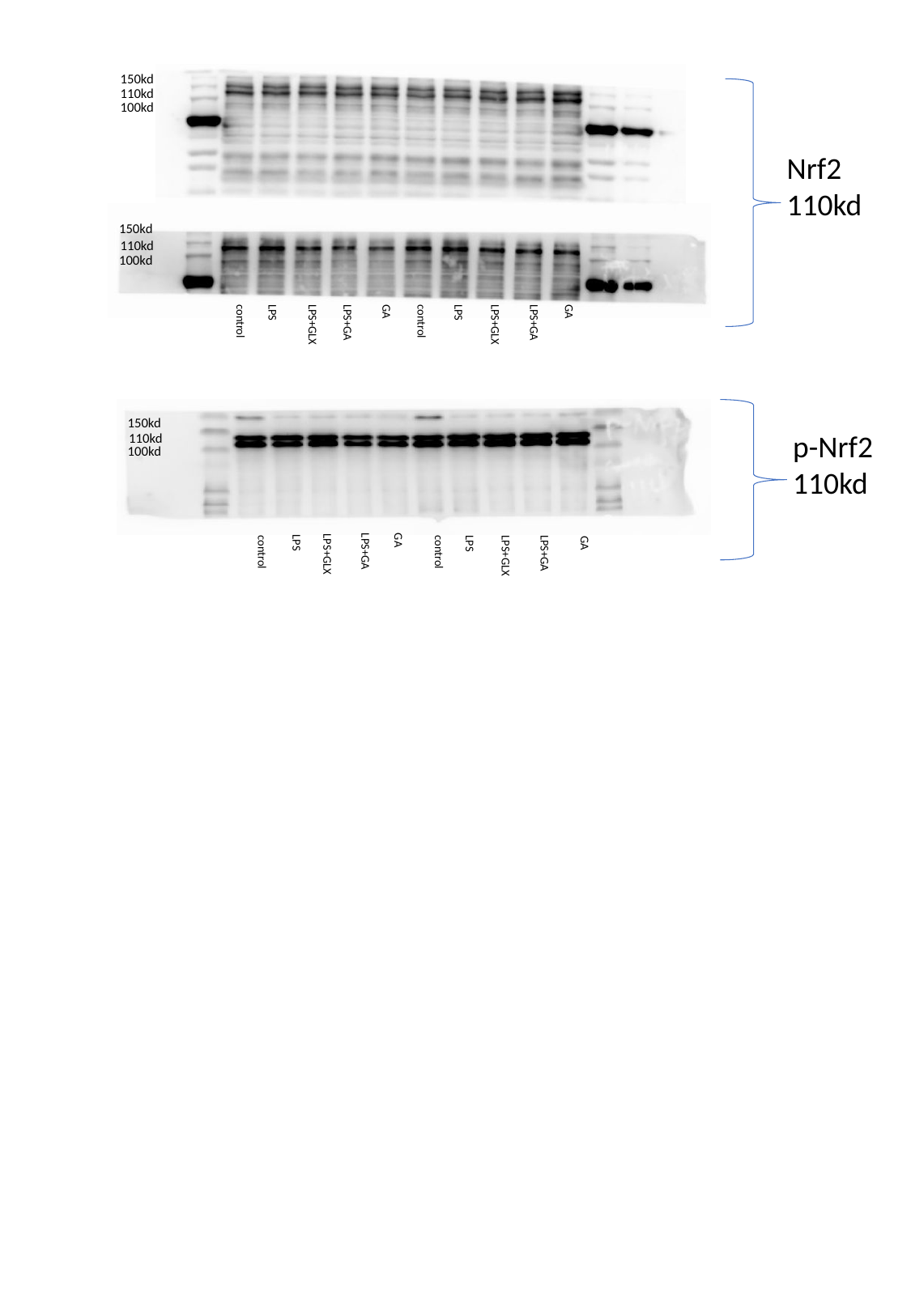

150kd
110kd
100kd
Nrf2
110kd
150kd
110kd
100kd
control
LPS
LPS+GLX
LPS+GA
GA
control
LPS
LPS+GLX
LPS+GA
GA
150kd
p-Nrf2
110kd
110kd
100kd
LPS+GA
GA
LPS+GLX
LPS
LPS+GA
control
control
LPS
LPS+GLX
GA

## Slide 4
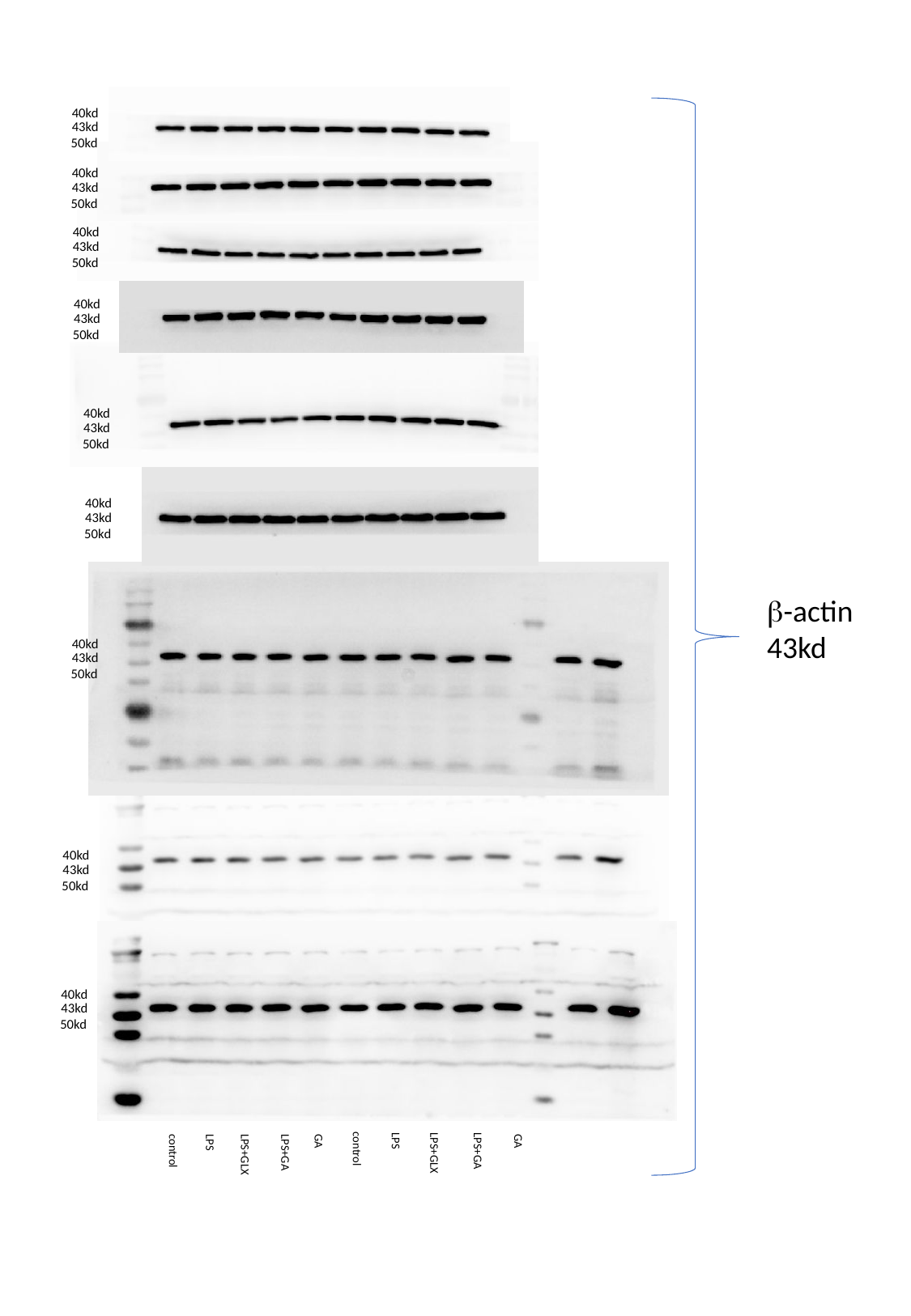

40kd
43kd
50kd
40kd
43kd
50kd
40kd
43kd
50kd
40kd
43kd
50kd
40kd
43kd
50kd
40kd
43kd
50kd
β-actin
43kd
40kd
43kd
50kd
40kd
43kd
50kd
40kd
43kd
50kd
control
LPS
LPS+GLX
LPS+GA
control
LPS
LPS+GLX
LPS+GA
GA
GA
